# Micro-Ultrasound in the Detection of Clinically Significant Prostate Cancer: A Comprehensive Review and Comparison with Multiparametric MRI

**DOI:** 10.3390/tomography11070080

**Published:** 2025-07-08

**Authors:** Julien DuBois, Shayan Smani, Aleksandra Golos, Carlos Rivera Lopez, Soum D. Lokeshwar

**Affiliations:** 1 Department of Urology, Yale School of Medicine, New Haven, CT 06510, USA; 2 The James Buchanan Brady Urological Institute and Department of Urology, Johns Hopkins University School of Medicine, Baltimore, MD 21287, USA

**Keywords:** micro-ultrasound, Exact-Vu, prostate cancer, prostate biopsy, PRI-MUS

## Abstract

Background/Objectives: Multiparametric MRI (mpMRI) is widely established as the standard imaging modality for detecting clinically significant prostate cancer (csPCa), yet it can be limited by cost, accessibility, and the need for specialized radiologist interpretation. Micro-ultrasound (micro-US) has recently emerged as a more accessible alternative imaging modality. This review evaluates whether the evidence base for micro-US meets thresholds comparable to those that led to MRI’s guideline adoption, synthesizes diagnostic performance data compared to mpMRI, and outlines future research priorities to define its clinical role. Methods: A targeted literature review of PubMed, Embase, and the Cochrane Library was conducted for studies published between 2014 and May 2025 evaluating micro-US in csPCa detection. Search terms included “micro-ultrasound,” “ExactVu,” “PRI-MUS,” and related terminology. Study relevance was assessed independently by the authors. Extracted data included csPCa detection rates, modality concordance, and diagnostic accuracy, and were synthesized and, rarely, restructured to facilitate study comparisons. Results: Micro-US consistently demonstrated non-inferiority to mpMRI for csPCa detection across retrospective studies, prospective cohorts, and meta-analyses. Several studies reported discordant csPCa lesions detected by only one modality, highlighting potential complementarity. The recently published OPTIMUM randomized controlled trial offers the strongest individual-trial evidence to date in support of micro-US non-inferiority. Conclusions: Micro-US shows potential as an alternative or adjunct to mpMRI for csPCa detection. However, additional robust multicenter studies are needed to achieve the evidentiary strength that led mpMRI to distinguish itself in clinical guidelines.

## 1. Introduction

Prostate cancer is the most commonly diagnosed non-cutaneous malignancy among men and remains the second leading cause of cancer-related death, accounting for an estimated 35,000 deaths in 2024 [1]. The accurate identification of clinically significant prostate cancer (csPCa) is therefore critical to ensure appropriate treatment while avoiding the overtreatment of indolent tumors. Histopathologic evaluation from prostate biopsy remains the gold standard for the risk stratification of prostate cancer [2]. Historically, transrectal ultrasound (TRUS) without accompanying MRI was the primary modality for targeted prostate biopsies, but poor lesion visualization limited its accuracy [3]. Consequently, systematic biopsy protocols remained standard practice.

This paradigm began to shift with the publication of the PROMIS trial in 2017, which demonstrated that multiparametric MRI (mpMRI) offered superior sensitivity for csPCa compared to TRUS biopsy [4]. Subsequent trials and meta-analyses supported the fact that mpMRI significantly increased diagnostic yield compared to systematic biopsy alone [5]. As a result, contemporary clinical guidelines recommend the incorporation of prostate MRI in the standard-of-care pathway for biopsy-naïve men and men who have had a previously negative prostate biopsy [6,7]. Despite the evidence supporting its use, mpMRI is limited by a lack of machine accessibility, high costs, radiologist shortages, and absolute and relative patient contraindications such as select implants and claustrophobia [8,9,10,11].

In response to mpMRI’s limitations, centered around resource scarcity and patient contraindications, micro-ultrasound (micro-US) has emerged as a novel high-resolution imaging modality for prostate cancer detection. Unlike conventional ultrasound, which operates at 6–9 MHz, micro-US uses a 29 MHz transducer, offering real-time visualization of prostatic anatomy at near-histologic resolution. Unlike mpMRI, micro-US can be performed at the time of biopsy without the need for pre-procedural imaging, making it a more accessible and cost-effective potential diagnostic alternative.

Despite growing interest and adoption, there has been a paucity of comprehensive multicenter RCTs and paired studies with robust blinding which assess this novel imaging modality. Addressing this gap, the OPTIMUM trial, a Phase III multicenter RCT, was recently published, demonstrating that micro-US was non-inferior to mpMRI in the detection of csPCa [12].

Considering new developments, this review critically evaluates the existing and recently published body of evidence assessing micro-US as a diagnostic tool for csPCa. We contextualize the trajectory that led to mpMRI adoption in contemporary PCa guidelines to gauge whether micro-US meets similar evidentiary thresholds. We synthesize and compare diagnostic performance across modalities, highlight key areas of concordance and discordance in csPCa detection, and identify gaps in the current literature. Our aims are to explore whether the current evidence is sufficient to consider micro-US a viable alternative or complement to mpMRI, and to outline future research directions that could define its role in clinical algorithms.

## 2. Materials and Methods

A preliminary literature search revealed that the study by Pavlovich et al. (2014) was one of the first studies to evaluate high-frequency ultrasound for PCa detection, making 2014 a practical starting point for this review [13]. We subsequently conducted a targeted literature review of PubMed, Embase, and the Cochrane Library for peer-reviewed studies published between 2014 and May 2025 assessing the clinical performance and applicability of micro-US in PCa detection. Search terms included “micro-ultrasound,” “ExactVu,” “PRI-MUS,” and “high-frequency,” and additional relevant articles were identified using variations and related terminology not explicitly listed here.

To further capture relevant studies, we reviewed the manufacturer-curated bibliography hosted by Exact Imaging (https://www.exactimaging.com/papers-and-publications accessed on 18 April 2025). Studies from this source were included only if they were available as full-text, peer-reviewed publications and were indexed on PubMed, but may not have been captured through our initial database queries due to variable terminology.

Study inclusion was based on relevance to micro-US diagnostic performance or clinical utility and was assessed independently by members of the study team. Table 1 lists the studies that were used to assess micro-US. Both retrospective and prospective designs were considered. For each study, we extracted key information on sample size, study design, comparator modality, and outcomes related to csPCa detection and diagnostic accuracy. In rare cases, studies’ reported data were restructured to enable more direct comparison between studies. This was performed, for example, with results from Wiemer et al. (2021), which were reformatted to facilitate side-by-side comparison with results from Lughezzani et al. (2021) on the concordant and discordant detection of PCa lesions between mpMRI and micro-US [14,15].

## 3. Overview of Imaging Modalities

### 3.1. MRI Overview

MRI, introduced in the 1980s, uses strong magnetic fields to manipulate nuclear spins, producing signals that are mathematically reconstructed into detailed anatomical images [34]. Traditional T1- and T2-weighted sequences are used for structural imaging, while functional sequences such as diffusion-weighted imaging (DWI) and dynamic contrast-enhanced MRI (DCE-MRI) provide physiological information, such as water molecule diffusion and vascular contrast uptake [35]. Multiparametric MRI combines these structural and functional techniques to enhance the detection and characterization of prostate lesions. 

The Prostate Imaging–Reporting and Data System (PI-RADS) was first introduced by the European Society of Urogenital Radiology (ESUR) in 2012 to standardize mpMRI interpretation [36]. PI-RADS assigns scores from 1 to 5 based on the likelihood of representing csPCa, with criteria tailored to the imaging modality and, in some cases, the lesion location within the prostate. PI-RADS v2, released in 2015, improved protocol standardization and diagnostic clarity, resulting in enhanced sensitivity while maintaining comparable specificity [37,38]. The most recent iteration, PI-RADS v2.1 (2019), was developed to improve reader consistency, although the current literature suggests no significant diagnostic performance difference compared to v2 [39,40].

In the late 2010s, a series of seminal multicenter prospective studies listed in Table 2 helped redefine the role of mpMRI in prostate cancer diagnosis. The PROMIS study (2017) demonstrated that mpMRI had substantially higher sensitivity than TRUS biopsy for detecting csPCa when both were benchmarked against template mapping biopsy, while also reducing unnecessary biopsies [4]. The PRECISION randomized controlled trial (2018) reported that MRI-targeted biopsy detected more csPCa than systematic TRUS biopsy and diagnosed fewer cases of clinically insignificant prostate cancer (GG1 PCa) [41]. Similarly, a prospective multicenter study by van der Leest et al. (2019) found that mpMRI and TRUS had comparable csPCa detection abilities, but mpMRI resulted in significantly fewer diagnoses of clinically insignificant cancer, supporting its value in reducing overtreatment [42]. The MRI-FIRST study (2019) corroborated these findings, showing that combining MRI-targeted with systematic biopsy provided superior detection rates [43].

The cumulative influence of these studies culminated in a major shift in the 2023 AUA guidelines, which now recommend incorporating mpMRI and PI-RADS, either with or without systematic biopsy, in the diagnostic pathway for suspected PCa [6]. This marked a significant change from the 2018 AUA guidelines, which did not differentiate between TRUS, MRI, and biomarker-based diagnostics, and helped to solidify mpMRI’s role as the standard of care in many practice settings [44].

### 3.2. Micro-Ultrasound Overview

Exact Imaging’s ExactVu 29 MHz micro-US system is currently the only clinically studied micro-US platform for the detection of csPCa, with its first adoption by a health system in 2017 [45,46]. Micro-US offers potential advantages over conventional TRUS due to its significantly higher operating frequency [13,16]. This increase in frequency has direct implications for image resolution, a key determinant of lesion detection. Ultrasound resolution refers to the minimum distance at which two adjacent points can be distinguished and improves with increased frequency; however, this comes at this expense of imaging depth [47].

Micro-US’s higher frequency and optimized beam characteristics reportedly result in a 300% improvement in resolution, from approximately 200 μm to 70 μm, compared to conventional TRUS [16,24]. This resolution gain is sufficient to visualize individual prostatic ducts and finer architectural details. The system’s functional depth is limited to approximately 5–6 cm, a tradeoff generally deemed acceptable for PCa detection given that most csPCa arises in the peripheral zone, which lies closest to the rectal wall and therefore within ideal imaging range [21,48].

## 4. Micro-Ultrasound Clinical Use

### 4.1. Development of PRI-MUS

**Figure 1:**
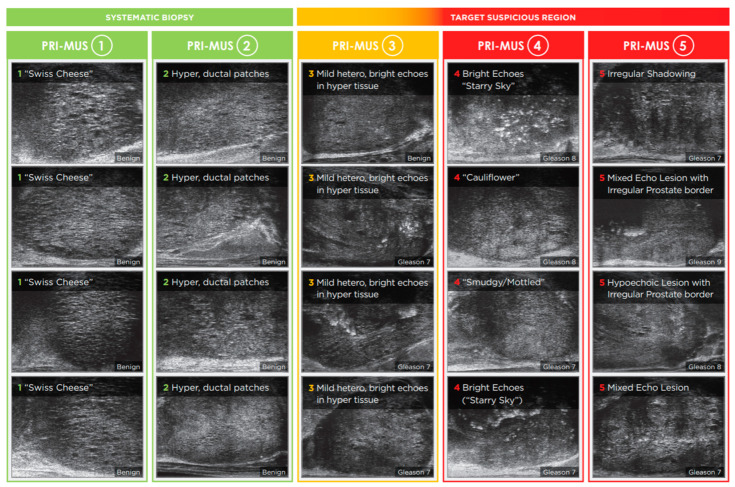
PRI-MUS protocol. Reproduced with permission from Exact Imaging. Biopsies of PRI-MUS 3-5 features correlate with increased rates of csPCa.

Recognizing the pivotal role PI-RADS played in distinguishing mpMRI from other prostate cancer imaging modalities, a parallel protocol was developed for micro-US to support similar clinical adoption. In 2016, the Prostate Risk Identification using Micro-US (PRI-MUS) system was introduced and validated for the peripheral zone, demonstrating a sensitivity of 80% and specificity of 37% for detecting csPCa in targeted lesions [16,19]. Compared to PI-RADS, PRI-MUS is a simpler risk-scoring system that uses standardized reference images to guide the interpretation of ultrasound features associated with varying levels of malignancy risk. The visual features of this protocol can be seen in Figure 1.

A subsequent update, PRI-MUS v2, was released in 2023 which expanded the protocol to include criteria for anterior zone lesions, offering guidance on both high- and low-risk imaging characteristics [49,50]. Currently, no data has been published which externally validates the anterior zone PRI-MUS protocol [33,50].

### 4.2. Micro-US vs. TRUS and Systematic Biopsy

As Table 3 displays, the initial studies which largely compared micro-US to conventional low-frequency TRUS suggested promising diagnostic advantages. In a pilot study by Pavlovich et al. (2014), higher-frequency ultrasound (16–21 MHz) was evaluated in patients with known prostate cancer and demonstrated a significant improvement over conventional lower-frequency ultrasound in the detection of any prostate cancer, with a nearly twofold increase in sensitivity (65% vs. 38%) and a higher detection rate (79% vs. 51%; *p* < 0.008) [13]. Although the detection of csPCa was also higher with high-frequency ultrasound (84% vs. 60%), the difference did not reach statistical significance (*p* = 0.11) with a sample size of 25.

Building on these preliminary findings, Eure et al. (2019) conducted a prospective head-to-head evaluation in a small cohort of nine patients, comparing micro-US to both mpMRI and conventional TRUS [17]. Micro-US demonstrated significantly greater sensitivity than TRUS (56–89% vs. 11%; *p* = 0.02) and successfully identified all three cases of Gleason Grade Group ≥2 cancer (100% vs. 33%). Abouassaly et al. (2020) also reported an improvement in csPCa detection when transitioning from conventional TRUS to micro-US (45% vs. 57%), although this was not statistically significant (*p* < 0.09) [19].

Contrary to the conclusions of prior studies, Pavlovich et al. (2021) conducted a multi-institutional RCT involving 1676 patients, finding no statistically significant difference between micro-US and conventional TRUS in overall csPCa detection (34.6% vs. 36.6%, *p* = 0.21) [25]. Both modalities demonstrated improved detection following PRI-MUS protocol training midway through the study. These findings supported the efficacy of PRI-MUS as a lesion risk-scoring protocol for micro-US but muddied the overall picture on micro-US. There are several important caveats to note regarding this study. Patients underwent systematic biopsy in addition to their assigned targeted biopsy modality, and an unreported higher csPCa detection rate for micro-US-targeted biopsies was found, indicating less reliance on systematic biopsy. The study used an outdated micro-US probe which has since been improved upon, and lastly, the protocol limited micro-US to 1 core/target compared to the 3–4 cores used in conventional ultrasound.

In a similar vein, Claros et al. (2020) found that while micro-US has a theoretically improved csPCa detection rate over the conventional ultrasound used in mpMRI fusion for targeted biopsies (38% vs. 23%, *p* = 0.02), their overall csPCa detection rates were not significantly different due to the added systematic biopsy coverage (40% vs. 32%, *p* = 0.24) [20].

Finally, the first meta-analysis by Zhang et al. (2019) looked at the accuracy of micro-US using pooled data from 769 patients and seven studies to estimate a sensitivity of 91% and specificity of 49% (AUC: 0.82) for csPCa detection, establishing early aggregate support for the technology [18]. While the specificity remains a relative limitation, the high sensitivity supports micro-US as a viable alternative to conventional TRUS, especially in targeted biopsy contexts. A second meta-analysis, that of Dariane et al.(2023), found that micro-ultrasound had comparable csPCa detection rates to systematic biopsy but detected statistically significantly fewer GG1 PCa cases than systematic biopsy [31].

### 4.3. Micro-US vs. mpMRI

As micro-US has matured beyond initial comparisons with conventional TRUS, a growing body of prospective studies and systematic reviews has evaluated its performance relative to mpMRI. In one of the earliest prospective comparisons, Eure et al. (2019) reported statistically insignificant micro-US superiority over mpMRI in the sensitivity of csPCa detection (56–89% vs. 33–56%, *p* = 0.37), with both outperforming conventional TRUS (11%) in a small cohort of nine patients [17].

Cornud et al. (2020) found that while 79% of suspicious lesions were visible on both modalities, among the discordant lesions, nearly a quarter (4/17) of the lesions only detected by micro-US were csPCa [21]. In contrast, none of mpMRI’s discordant lesions (0/13) were csPCa. Following Cornud et al. (2020) and Lughezzani et al. (2021), Wiemer et al. (2021) analyzed the concordance and discordance of lesion grades between mpMRI and micro-US [14,15,21]. Unlike the earlier study, these two found that mpMRI detected csPCa cases that micro-US did not, with micro-US and mpMRI each having instances of independently finding csPCa. While these results showcase the added potential of using the two technologies together in a complementary fashion, it should not be ignored that systematic biopsy detected 10% and 4% of csPCa cases that were missed by both modalities in Lughezzani et al. (2021) and Wiemer et al. (2021), respectively [14,15]. Table 4 summarizes the degrees of concordance and discordance in the mentioned studies.

In a prospective single-center paired diagnostic study using transperineal biopsies, Socarrás et al. (2020) found that PRI-MUS scoring outperformed PI-RADS in sensitivity (99.7% vs. 84.3%, *p* < 0.001) and negative predictive value (99.2% vs. 64.5%, *p* < 0.001), while specificity and positive predictive value (PPV) were comparable [22]. Notably, out of 194 patients with suspected PCa, the number of csPCa cases detected by a single modality and missed by the others were 11 for micro-US, 8 for systematic biopsy, and just 1 for mpMRI. Ghai et al. (2022) conducted a single-institution paired diagnostic trial, further advocating for micro-US’s clinical equivalency [27]. Ghai’s single-institution trial reported similar csPCa detection rates between mpMRI and micro-US, with MRI avoiding more biopsies but not detecting more csPCa.

To date, there have been four prospective multicenter studies assessing micro-US against mpMRI, as seen in Table 5. In the first of these multicenter studies, Klotz et al. (2021) reported that micro-US had significantly higher sensitivity over mpMRI (94% vs. 90%, *p* = 0.03), and specific non-inferiority analysis concluded non-inferiority (*p* < 0.001) for sensitivity, specificity, negative predictive value (NPV), and PPV between the imaging modalities for both GG ≥ 2 and GG ≥ 3 [24]. Non-uniform study site protocols and inconsistent blinding reduce the generalizability of this study, with 9 out of the 11 sites being unblinded, representing 71% of the study population.

Hofbauer et al. (2022) conducted a study in Germany and Austria, also supporting the non-inferiority between the imaging tools for the detection of csPCa (*p* = 0.02) [28]. Micro-US and mpMRI alone found 6% and 9% of csPCa cases, suggesting the potential for complementary use. Patients were excluded if their known prostate volume was greater than 100 mL, potentially limiting the generalizability of this study.

Further making the case for micro-US, Rojo et al. (2024) found no differences in csPCa detection rates between the modalities and concluded that micro-US “appears to be an equally relevant diagnostic method” to mpMRI fusion biopsies for the detection of csPCa [32]. This conclusion, however, seems overstated. The study failed to detect statistically significant differences in csPCa detection (21.25% vs. 18.75%, *p* = 0.45) or overall cancer detection (50% vs. 51.25%, *p* = 0.09) between mpMRI and micro-US, respectively, but did not perform a formal non-inferiority analysis. A letter to the editor, authored by researchers with ties to the MRI fusion company Koelis, highlights other potential study limitations [51].

The development and dissemination of these comparative studies prompted multiple meta-analyses to comprehensively assess micro-US’s diagnostic performance against MRI. Three meta-analyses from 2021 to 2023, those by Sountoulides et al. (2021), You et al. (2022), and Cotter et al. (2023), all found that no significant differences existed between mpMRI and micro-US for csPCa detection, reinforcing the case for the clinical use of micro-US [26,29,30]. Table 6 summarizes the findings from these meta-analyses.

Finally, the largest and most rigorously designed comparison between micro-US and MRI to date, OPTIMUM, found that micro-US, mpMRI/micro-US fusion, and mpMRI fusion were all non-inferior for both single-modality-targeted csPCa detection and overall csPCa detection with added systematic biopsy [12]. Solo systematic biopsy underperformed compared to all three, and 4.4% of patients had csPCa detected by only one modality, again highlighting the potential benefit of combined imaging strategies. OPTIMUM provides the strongest evidence yet for true micro-US non-inferiority to mpMRI, and is arguably the only micro-US study at a comparable quality to MRI’s landmark studies.

## 5. Discussion

The growing body of evidence evaluating micro-US for prostate cancer detection suggests that it may be a promising alternative or complement to mpMRI for the diagnosis of PCa. Systematic reviews and meta-analyses, as well as the recently published OPTIMUM trial, support its non-inferiority to mpMRI in both targeted and overall csPCa detection. All three micro-US/MRI systematic reviews have found no significant differences between csPCa and GG1 PCa detection rates based on the current literature.

Within the landmark OPTIMUM trial, there were no significant differences in GG ≥ 2 or GG ≥ 3 detection rates between imaging modalities. PI-RADS and PRI-MUS were also both found to have no significant differences in NPV for scores less than three, which indicate low likelihoods of csPCa. However, the overall strength of micro-US’s prospective multicenter studies continues to fall short compared to what existed for mpMRI when its standing in clinical guidelines was changed.

Importantly, prospective data and inherent technology differences raise the possibility that micro-US and mpMRI may identify different subsets of csPCa, with studies repeatedly reporting that each image modality detects a small fraction of csPCa cases missed by the other. While these differences were often statistically insignificant, they may be clinically significant when considering the impact that any missed csPCa has on patient outcomes. This evidence base supports the rationale that a combined diagnostic strategy may enhance overall csPCa detection, potentially diminishing the importance of concluding micro-US as a strict adjunct to MRI. Specifically, mpMRI–micro-US fusion imaging, augmented by systematic biopsy, may offer the most comprehensive approach for patients with clinical suspicion of prostate cancer.

Clinically, study evidence supporting micro-US non-inferiority is highly relevant. While mpMRI remains the current standard of care, its accessibility can be limited by its high cost, longer scheduling times, and the need for specialized radiologic interpretation. Contraindications to MRI, such as certain implants or severe claustrophobia, further restrict its use. In contrast, micro-US offers real-time lesion visualization at the point of care and can be utilized without the need for separate pre-procedural imaging or coordination with radiology services. These characteristics make micro-US a more accessible and cost-effective tool, particularly in resource-limited settings or community-based urologic practices.

### Limitations and Challenges of Micro-US

Micro-US faces several notable limitations and implementation challenges that limit its wider adoption. Like mpMRI, micro-US is highly operator-dependent. Rosenkrantz et al. (2016) suggest that novice radiologists require 40 cases before PI-RADS proficiency begins to plateau, while on the operator side it appears that urologists require 100–110 cases to be proficient with transrectal TRUS/MRI fusion biopsies [52,53,54]. A retrospective study by Cash et al. (2022) suggests that it takes 20–40 cases and 40–90 cases for a micro-US operator who is already experienced with fusion biopsy to achieve expert-level sensitivity and specificity through Exact Imaging’s formal training program [55]. To our knowledge, no studies exist that assess the PRI-MUS/micro-US learning curve for urologists with minimal TRUS experience. This gap in our knowledge limits the ability to directly compare the learning curves since it is unclear to what extent TRUS experience translates to micro-US.

The widespread adoption of micro-US would also likely be faced with logistical challenges, at least in the short term. Micro-US’s success as a cost-effective and accessible alternative to MRI would foreseeably strain Exact Imaging’s current supply chains and production capabilities. While it appears that Exact Imaging is able to meet the current demand, any inability to adequately deliver and support their product would certainly limit micro-US adoption and utilization since there is no alternative micro-US supplier at this time.

The disparity in professional society recognition of PI-RADS and PRI-MUS is also important to highlight. As stated by Weinreb et al. (2016), PI-RADS v2 was designed by the American College of Radiology, European Society of Uroradiology, and Admetech Foundation, in addition to the AUA incorporating PI-RADS into current PCa detection guidelines [6,37]. The widespread professional society recognition of PRI-MUS is comparatively absent. This absence of recognition adds to the current challenge of micro-US integration into clinical decision-making.

Evidence remains mixed regarding inter-reader variability in lesion characterization [33]. Unlike asynchronous PI-RADS interpretations, which allow radiologists to consult colleagues or seek second opinions before finalizing reports, PRI-MUS scoring is performed in real-time during micro-US-guided biopsy. This aspect of micro-US may amplify the clinical impact of inter-reader variability, even if variability rates are ultimately comparable to those seen with PI-RADS.

Longitudinal monitoring and active surveillance are also not well validated for micro-US. Unlike mpMRI, which produces archivable, standardized images that enable the objective tracking of lesions over time, micro-US is largely interpreted in real-time and lacks established protocols for image storage or reproducible lesion localization, limiting its role in long-term follow up.

Micro-US’s intrinsic technological characteristics of having limited imaging depth may also pose a challenge for its widespread adoption [21]. The prospective database compiled by Chessa et al. (2021) found that a significant drop in sensitivity and AUC was seen in the anterior/transition zone (sensitivity: 45%; AUC: 0.54) in relation to the peripheral zone (sensitivity: 74%; AUC: 0.75) [23]. Zhou et al. (2024) conducted a prospective multicenter study assessing inter-reader agreement and also found a significant decrease in csPCa detection in the anterior gland, with six out of the nine anterior gland lesions being missed by all six of the urologist readers [33]. Chessa et al. (2021) predated the development of an anterior gland PRI-MUS protocol, while it is implied that this protocol was used by Zhou et al. (2024), though not explicitly stated [23,33]. At the time of this review, the anterior gland PRI-MUS protocol remains unvalidated by an external study.

Another significant challenge is the current evidence base for micro-US, which remains less mature and rigorous than that of mpMRI. While there have been several large-scale prospective multicenter clinical trials evaluating mpMRI, the OPTIMUM trial and perhaps the study by Hofbauer et al. (2022) appear to be the only micro-US multicenter prospective studies to have rigorous study designs without significant generalizability limitations [12,28]. Pavlovich et al. (2021) display the efficacy of PRI-MUS, but ultimately fail to report micro-US superiority over TRUS [25]. However, the study has several design limitations unfavorable to micro-US, reducing the trial’s current generalizability. The other prospective multicenter studies, those by Klotz et al. (2021) and Rojo et al. (2024), had limitations relating to blinding and study analysis, respectively [24,32].

## 6. Future Directions

### 6.1. Clinical Validation and Integration into Guidelines

Recent research has identified several potential paths for the widespread adoption and integration into prostate cancer guidelines of micro-US technology. Future efforts should prioritize head-to-head, multicenter studies with robust study design that compare micro-US with mpMRI or mpMRI-micro-US fusion imaging, not only to validate the results of the OPTIMUM trial but also to evaluate the performance of micro-US in patients with prior negative biopsy results, those undergoing active surveillance, or those who have completed treatment. Of note, there are two ongoing multicenter trials led by the University of Alberta which are projected to contribute evidence relating to OPTIMUM’s validation and the utility of micro-US for csPCa active surveillance [56,57]. Lastly, there remains a need to externally validate the anterior gland PRI-MUS protocol [33,50].

Large multicenter prospective trials such as these are needed to validate the findings of the OPTIMUM trial and establish micro-US as part of American Urological Association and European Association of Urology guidelines, a trajectory that mirrors the path mpMRI followed after the publication of the PROMIS, PRECISION, van der Leest, and MRI-FIRST trials.

### 6.2. Surgical Planning and Tumor Staging

Future research should explore opportunities for micro-US to enhance surgical planning and pre-op staging. Regis et al. (2020) first evaluated micro-US features predictive of extracapsular extension (ECE), identifying four derived from MRI and one unique to micro-US [58]. Fasulo et al. (2022) validated these features prospectively prior to radical prostatectomy, finding concordance between micro-US predictions and final pathology in 81.4% of patients [59].

If it is discovered that micro-US does indeed systematically under-detect anterior lesions, additional research may find potential in developing workarounds for this limitation. Potential solutions include a complementary transurethral micro-US approach via cystoscope and dual-frequency probes that allow the operator to seamlessly sacrifice some frequency in exchange for increased effective depth [60,61].

Another potential clinical application of micro-US is the prediction of pathological tumor volume in prostate cancer lesions. Richemond et al. (2025) conducted a retrospective comparison of micro-US and MRI in predicting tumor volume on final pathology [62]. Micro-US and MRI were found to underpredict tumor volume by 0.15 mL and 0.26 mL, respectively. Future work should assess the ability of micro-US to aid in decision-making in settings such as nerve-sparing surgery, pelvic lymph node dissection, and focal prostate lesion treatment.

### 6.3. Artificial Intelligence and Operator Standardization

Finally, there are several promising directions to improve the accuracy and reliability of micro-US in prostate cancer diagnosis. Artificial intelligence (AI) and machine learning (ML) algorithms have been widely used to help detect and classify suspicious lesions on MRI or conventional ultrasound and facilitate fusion biopsies [63]. Studies by Rohrbach et al. (2018) and Shao et al. (2020) suggest that AI can improve lesion classification and operator AUC, while Jiang et al. (2024) demonstrated improved segmentation in challenging prostate anatomies [64,65,66]. These proof-of-concept findings support the future integration of AI to augment micro-US and improve clinical outcomes.

Future efforts should also seek to address concerns about operator dependence in performing micro-US imaging and inter-reader variability in characterizing lesions [33]. This may be accomplished by leveraging AI and ML methods, further refining the PRI-MUS system, or reassessing the existing formal micro-US training program’s ability to standardize reader interpretations [55].

## 7. Conclusions

While micro-ultrasound is not yet established in clinical guidelines, accumulating evidence supports its potential as a viable alternative or adjunct to mpMRI for the detection of csPCa during image-guided prostate biopsy. Additional robust multicenter studies are needed to validate the recent OPTIMUM trial, anterior gland PRI-MUS protocol, and to achieve the overall evidentiary strength that led mpMRI to distinguish itself in clinical guidelines.

## Figures and Tables

**Table 1:** Summary of clinical studies evaluating micro-ultrasound for prostate cancer detection.

Study	Enrollment Years	N	Study Type	Patient Population (BN = Biopsy-Naïve, AS = Active Surveillance)	Analysis Focus	Key Findings
Pavlovich 2014 [13]	2010–2011	25	Prospective (Pilot Study)	100% Biopsy-Proven PCa	Lesion	Higher-frequency (16–21 MHz) displayed promising, but mostly insignificant improvements over lower-frequency US
Ghai 2016 [16]	2013–2016	100 Cines	Prospective Collection w/Retrospective Diagnostic Validation	0% AS, Unknown BN	Lesion	PRI-MUS protocol displays potential to be comparable to PI-RADS
Eure 2019 [17]	16 December	9	Prospective Paired Diagnostic	100% AS	Patient	Micro-US and mpMRI clinically comparable, while both outperformed conventional US
Zhang 2019 [18]	NA *	769	Systematic Review	Mixed	Pooled	First micro-US meta-analysis establishing pooled data; sensitivity: 0.91; specificity: 0.49; AUC: 0.82
Abouassaly 2020 [19]	January–August 2018	67	Prospective Paired Diagnostic	72% BN	Patient	Transition from conventional US to micro-US led to relatively increased csPCa detection
Claros 2020 [20]	February 2017–September 2018	269	Retrospective Cohort	26% BN	Patient	Overall DR not statistically different, but targeted micro biopsies had significantly higher detection rates
Cornud 2020 [21]	February–June 2019	118 Patients, 144 Lesions	Prospective Paired Diagnostic	67% BN, 18% AS	Mixed	mpMRI and micro-US both had instances of missed lesions, but only mpMRI missed csPCa lesions
Socarras 2020 [22]	February 2018–September 2019	194	Prospective Paired Diagnostic	66% BN	Patient	PRI-MUS had statistically significantly improved sensitivity and NPV over PI-RADS
Chessa 2021 [23]	June–September 2018	83	Prospective Paired Diagnostic	100% Biopsy-Proven PCa	Patient	Micro-US sensitivity considerably worse for anterior/transition zone lesions compared to peripheral zone
Klotz 2021 [24]	NA *	1040	Prospective Multicenter Paired Diagnostic	66% BN, 6.3% AS	Patient	Micro-US sensitivity superior to mpMRI with non-inferior specificity
Lughezzani 2021 [15]	October 2017–September 2019	320	Prospective Paired Diagnostic	63% BN	Patient	mpMRI and micro-US roughly equivocal; systematic biopsy found 10.3% of csPCa missed by targeted biopsy
Pavlovich 2021 [25]	2013–2016	1676	Prospective Multicenter RCT	0% AS, Unknown BN	Patient	No statistically significant difference between micro-US and conventional US; both saw improvements after PRI-MUS training
Sountoulides 2021 [26]	NA *	1125	Systematic Review/Meta-Analysis	Unspecified	Patient	No significant differences in mpMRI and micro-US for detection of GG ≥ 2 or GG ≥ 3 csPCa
Wiemer 2021 [14]	February-December 2018	159	Prospective Paired Diagnostic	45% BN, 29% AS	Patient	mpMRI and micro-US fusion superior modality; suggests elimination of systematic biopsy
Ghai 2022 [27]	May 2019–September 2020	94	Prospective Paired Diagnostic	100% BN	Patient	Comparable mpMRI and micro-US csPCa detection rates, with mpMRI preventing more biopsies; no added value of systematic biopsy to mpMRI/micro-US fusion
Hofbauer 2022 [28]	January–December 2019	203	Prospective Multicenter Paired Diagnostic	72% BN	Patient	Micro-US discovered 97% of csPCa detected by mpMRI; micro-US non-inferior to mpMRI
You 2022 [29]	NA *	1081	Systematic Review/Meta-Analysis	Unspecified	Patient	No significant differences in mpMRI and micro-US for detection of GG = 1, GG ≥ 2, or GG ≥ 3 csPCa
Cotter 2023 [30]	NA *	1759	Systematic Review/Meta-Analysis	0% AS, Unknown BN	Patient	No statistically significant difference in sensitivity or specificity between micro-US and mpMRI
Dariane 2023 [31]	NA *	2967	Systematic Review/Meta-Analysis	Unspecified	Patient	Micro-US outperforms systematic biopsy with higher detection of csPCa and lower non-clinically significant PCa
Rojo 2024 [32]	May 2021–June 2022	80	Prospective Multicenter Paired Diagnostic	0% AS, Unknown BN	Patient	No significant difference between micro-US and mpMRI detected
Zhou 2024 [33]	2022–2023	56 Patients	Prospective Collection w/Retrospective Diagnostic Validation	74% BN, 21% AS	Lesion	All six urologists agreed on lesion only 33% of the time; lowest sensitivity and agreement found with anterior prostate lesions
Kinnaird (OPTIMUM) 2025 [12]	December 2021–September 2024	678	Prospective Multicenter RCT	100% BN	Patient	Solo micro-US, mpMRI and micro-US fusion, and conventional mpMRI fusion all non-inferior for csPCa detection; solo systematic biopsy was inferior to all modalities for detection of csPCa

* Total enrollment periods not reported within the study.

**Table 2:** Sensitivity, specificity, and predictive values of MRI vs. TRUS in landmark csPCa detection trials.

Study	N	Definition of csPCa	Sensitivity		Specificity		PPV		NPV	
			MRI	TRUS	MRI	TRUS	MRI	TRUS	MRI	TRUS
PROMIS (2017) [4]	576	GG ≥ 3 or cancer core length ≥6 mm	93% *	48%	41%	96% *	51%	90% *	89% *	74%
		GG ≥ 2 or cancer core length ≥4 mm	87% *	60%	47%	98% *	69%	98% *	72% *	65%
		GG ≥ 2	88% *	48%	45%	99% *	65%	99% *	76% *	63%
			**csPCa Detection Rate**		**GG1** **PCa DR**					
			**MRI**	**TRUS**	**MRI**	**TRUS**				
PRECISION (2018) [41]	500	GG ≥ 2	38% *	26%	9% *	22%	–	–	–	–
Van der Leest (2019) [42]	626	GG ≥ 2	25%	23%	14%	25% *	–	–	–	–
MRI-FIRST (2019) [43]	251	GG ≥ 2	32%	30%	6%	20% *	–	–	–	–

* denotes significance.

**Table 3:** Micro-ultrasound vs. conventional TRUS: csPCa detection rates, sensitivity, and NPV.

Study	N	Population (BN = Biopsy-Naïve, AS= Active Surveillance)	csPCa Detection Rate		Any PCa DR		Sensitivity		NPV	
			Micro	Conv	Micro	Conv	Micro	Conv	Micro	Conv
Pavlovich (2014) [13]	69	100% Biopsy-Proven PCa	65%	38%	–	–	65%	38%	71%	55%
Eure (2019) [17]	9	100% Active Surveillance	33%	11%	78%	22%	56–89%	11%	96–98%	93%
Abouassaly (2020) [19]	67	72% Biopsy-Naïve	57%	45%	–	–	–	–	–	–
Claros (2020) [20]	222	26% Biopsy-Naïve	40%	32%	68%	64%	–	–	–	–
Pavlovich (2021) [25]	1676	0% AS, Unknown BN	35%	37%	50%	54%	61%	38%	92%	92%

**Table 4:** Concordance and discordance between micro-ultrasound and mpMRI in detection of clinically significant prostate cancer.

Study	N	Total Confirmed csPCa	Estimated Cores Taken Per Patient		Concordant Micro-US and mpMRI PCa	No. of Discordant csPCa Lesions or Cases		Confirmed csPCa Lesions or Cases among Discordant Targets		
			Targeted	Systematic		Micro	mpMRI	Micro	mpMRI	SB
Cornud (2020) [21]	144 Lesions	74	–	–	79% (114/144)	17	13	4	0	–
Lughezzani (2021) [15]	320 Patients	116	4	9	74% (189/255)	20	14	3	3	12
Wiemer (2021) [14]	159 Patients	78	8	8	44% (32/72)	27	13	20	1	3

**Table 5:** Summary of prospective multicenter studies assessing micro-US and MRI.

Micro-US Study	Study Design	No. Subjects Enrolled	No. Centers	Key Findings
Klotz (2021) [24]	Prospective Paired Diagnostic	1040	11	Micro-US sensitivity superior and specificity non-inferior to mpMRI
Hofbauer (2022) [28]	Prospective Paired Diagnostic	203	3	Micro-US non-inferior to mpMRI for detection of csPCa
Rojo (2024) [32]	Prospective Paired Diagnostic	80	4 *	No differences in csPCa detection found between micro-US and mpMRI
Kinnaird (OPTIMUM) (2025) [12]	RCT	802	20	Micro-US non-inferior to mpMRI for detection of csPCa

* Site count not specifically stated and assumed based on author institutions.

**Table 6:** Diagnostic performance ratios and pooled sensitivity/specificity from meta-analyses of micro-US vs. mpMRI.

Study	No. of Studies	No. of Patients	Micro-US: mpMRI csPCa Detection Ratio		Micro-US: mpMRI GG = 1 PCa Detection Ratio	
Sountoulides (2021) [26]	13	1125	1.05 (0.93–1.19)		0.94 (0.73–1.22)	
You (2022) * [29]	11	1081	1.01 (0.83–1.22)		0.92 (0.68–1.25)	
			**csPCa Sensitivity**		**csPCa Specificity**	
			**Micro**	**mpMRI**	**Micro**	**mpMRI**
Cotter (2023) [30]	12	1759	89% (83–93%)	86% (73–93%)	31% (23–40%)	32% (18–50%)

* You (2022) had a 91% study overlap with Sountoulides (2021).

## Data Availability

No new data were created or analyzed in this study.

## References

[1] Siegel R.L., Kratzer T.B., Giaquinto A.N., Sung H., Jemal A. (2025). Cancer statistics, 2025. CA Cancer J. Clin..

[2] Litwin M.S., Tan H.J. (2017). The Diagnosis and Treatment of Prostate Cancer: A Review. JAMA.

[3] Yacoub J.H., Verma S., Moulton J.S., Eggener S., Oto A. (2012). Imaging-guided Prostate Biopsy: Conventional and Emerging Techniques. RadioGraphics.

[4] Ahmed H.U., Bosaily A.E.S., Brown L.C., Gabe R., Kaplan R., Parmar M.K., Collaco-Moraes Y., Ward K., Hindley R.G., Freeman A. (2017). Diagnostic accuracy of multi-parametric MRI and TRUS biopsy in prostate cancer (PROMIS): A paired validating confirmatory study. Lancet.

[5] Drost F.J.H., Osses D.F., Nieboer D., Steyerberg E.W., Bangma C.H., Roobol M.J., Schoots I.G. (2019). Prostate MRI, with or without MRI-targeted biopsy, and systematic biopsy for detecting prostate cancer. Cochrane Database Syst. Rev..

[6] Early Detection of Prostate Cancer: AUA/SUO Guideline (2023)-American Urological Association. https://www.auanet.org/guidelines-and-quality/guidelines/early-detection-of-prostate-cancer-guidelines.

[7] Cornford P., van den Bergh R.C.N., Briers E., Van den Broeck T., Brunckhorst O., Darraugh J., Eberli D., De Meerleer G., De Santis M., Farolfi A. (2024). EAU-EANM-ESTRO-ESUR-ISUP-SIOG Guidelines on Prostate Cancer—2024 Update. Part I: Screening, Diagnosis, and Local Treatment with Curative Intent. Eur. Urol..

[8] Schoots I.G., Barentsz J.O., Bittencourt L.K., Haider M.A., Macura K.J., Margolis D.J.A., Moore C.M., Oto A., Panebianco V., Siddiquiet M.M. (2021). PI-RADS Committee Position on MRI Without Contrast Medium in Biopsy-Naive Men With Suspected Prostate Cancer: Narrative Review. Am. J. Roentgenol..

[9] Faria R., Soares M.O., Spackman E., Ahmed H.U., Brown L.C., Kaplan R., Emberton M., Sculpher M.J. (2018). Optimising the Diagnosis of Prostate Cancer in the Era of Multiparametric Magnetic Resonance Imaging: A Cost-effectiveness Analysis Based on the Prostate MR Imaging Study (PROMIS). Eur. Urol..

[10] Padhani A.R., Godtman R.A., Schoots I.G. (2024). Key learning on the promise and limitations of MRI in prostate cancer screening. Eur. Radiol..

[11] Ghadimi M., Thomas A. (2025). Magnetic Resonance Imaging Contraindications. StatPearls.

[12] Kinnaird A., Luger F., Cash H., Ghai S., Urdaneta-Salegui L.F., Pavlovich C.P., Brito J., Shore N.D., Struck J.P., Schostak M. (2025). Microultrasonography-Guided vs MRI-Guided Biopsy for Prostate Cancer Diagnosis: The OPTIMUM Randomized Clinical Trial. JAMA.

[13] Pavlovich C.P., Cornish T.C., Mullins J.K., Fradin J., Mettee L.Z., Connor J.T., Reese A.C., Askin F.B., Luck R., Epstein J.I. (2014). High-resolution transrectal ultrasound: Pilot study of a novel technique for imaging clinically localized prostate cancer. Urol. Oncol. Semin. Orig. Investig..

[14] Wiemer L., Hollenbach M., Heckmann R., Kittner B., Plage H., Reimann M., Asbach P., Friedersdorff F., Schlomm T., Hofbaue S. (2021). Evolution of Targeted Prostate Biopsy by Adding Micro-Ultrasound to the Magnetic Resonance Imaging Pathway. Eur. Urol. Focus.

[15] Lughezzani G., Maffei D., Saita A., Paciotti M., Diana P., Buffi N.M., Colombo P., Elefante G.M., Hurle R., Lazzeri M. (2021). Diagnostic Accuracy of Microultrasound in Patients with a Suspicion of Prostate Cancer at Magnetic Resonance Imaging: A Single-institutional Prospective Study. Eur. Urol. Focus.

[16] Ghai S., Eure G., Fradet V., Hyndman M.E., McGrath T., Wodlinger B., Pavlovich C.P. (2016). Assessing Cancer Risk on Novel 29 MHz Micro-Ultrasound Images of the Prostate: Creation of the Micro-Ultrasound Protocol for Prostate Risk Identification. J. Urol..

[17] Eure G., Fanney D., Lin J., Wodlinger B., Ghai S. (2019). Comparison of conventional transrectal ultrasound, magnetic resonance imaging, and micro-ultrasound for visualizing prostate cancer in an active surveillance population: A feasibility study. Can. Urol. Assoc. J..

[18] Zhang M., Wang R., Wu Y., Jing J., Chen S., Zhang G., Xu B., Liu C., Chen M. (2019). Micro-Ultrasound Imaging for Accuracy of Diagnosis in Clinically Significant Prostate Cancer: A Meta-Analysis. Front. Oncol..

[19] Abouassaly R., Klein E.A., El-Shefai A., Stephenson A. (2020). Impact of using 29 MHz high-resolution micro-ultrasound in real-time targeting of transrectal prostate biopsies: Initial experience. World J. Urol..

[20] Claros O.R., Tourinho-Barbosa R.R., Fregeville A., Gallardo A.C., Muttin F., Carneiro A., Stabile A., Moschini M., Macek P., Cathala N. (2020). Comparison of Initial Experience with Transrectal Magnetic Resonance Imaging Cognitive Guided Micro-Ultrasound Biopsies versus Established Transperineal Robotic Ultrasound Magnetic Resonance Imaging Fusion Biopsies for Prostate Cancer. J. Urol..

[21] Cornud F., Lefevre A., Flam T., Dumonceau O., Galiano M., Soyer P., Camparo P., Barral M. (2020). MRI-directed high-frequency (29MhZ) TRUS-guided biopsies: Initial results of a single-center study. Eur. Radiol..

[22] Socarrás M.E.R., Rivas J.G., Rivera V.C., Elbers J.R., González L.L., Mercado I.M., Del Alamo J.F., Del Dago P.J., Sancha F.G. (2020). Prostate Mapping for Cancer Diagnosis: The Madrid Protocol. Transperineal Prostate Biopsies Using Multiparametric Magnetic Resonance Imaging Fusion and Micro-Ultrasound Guided Biopsies. J. Urol..

[23] Chessa F., Schiavina R., Ercolino A., Gaudiano C., Giusti D., Bianchi L., Pultrone C., Marcelli E., Distefano C., Lodigiani L. (2021). Diagnostic accuracy of the Novel 29 MHz micro-ultrasound “ExactVuTM” for the detection of clinically significant prostate cancer: A prospective single institutional study. A step forward in the diagnosis of prostate cancer. Arch. Ital. Di Urol. E Androl..

[24] Klotz L., Lughezzani G., Maffei D., Sánchez A., Pereira J.G., Staerman F., Cash H., Luger F., Lopez L., Sanchez-Salas F. (2021). Comparison of micro-ultrasound and multiparametric magnetic resonance imaging for prostate cancer: A multicenter, prospective analysis. Can. Urol. Assoc. J..

[25] Pavlovich C.P., Hyndman M.E., Eure G., Ghai S., Caumartin Y., Herget E., Young J.D., Wiseman D., Caughlin C., Gray R.E. (2021). A multi-institutional randomized controlled trial comparing first-generation transrectal high-resolution micro-ultrasound with conventional frequency transrectal ultrasound for prostate biopsy. BJUI Compass.

[26] Sountoulides P., Pyrgidis N., Polyzos S.A., Mykoniatis I., Asouhidou E., Papatsoris A., Dellis A., Anastasiadis A., Lusuardi L., Hatzichristou D. (2021). Micro-Ultrasound–Guided vs Multiparametric Magnetic Resonance Imaging-Targeted Biopsy in the Detection of Prostate Cancer: A Systematic Review and Meta-Analysis. J. Urol..

[27] Ghai S., Perlis N., Atallah C., Jokhu S., Corr K., Lajkosz K., Incze P.F., Zlotta A.R., Jain U., Fleming H. (2022). Comparison of Micro-US and Multiparametric MRI for Prostate Cancer Detection in Biopsy-Naive Men. Radiology.

[28] Hofbauer S.L., Luger F., Harland N., Plage H., Reimann M., Hollenbach M., Gusenleitner A., Stenzl A., Schlomm T., Wiemer L. (2022). A non-inferiority comparative analysis of micro-ultrasonography and MRI-targeted biopsy in men at risk of prostate cancer. BJU Int..

[29] You C., Li X., Du Y., Peng L., Wang H., Zhang X., Wang A. (2022). The Microultrasound-Guided Prostate Biopsy in Detection of Prostate Cancer: A Systematic Review and Meta-Analysis. J. Endourol..

[30] Cotter F., Perera S., Sathianathen N., Lawrentschuk N., Murphy D., Bolton D. (2023). Comparing the Diagnostic Performance of Micro-Ultrasound-Guided Biopsy Versus Multiparametric Magnetic Resonance Imaging-Targeted Biopsy in the Detection of Clinically Significant Prostate Cancer: A Systematic Review and Meta-Analysis. Société Int. D’Urologie J..

[31] Dariane C., Ploussard G., Barret E., Beauval J.-B., Brureau L., Créhange G., Fromont G., Gauthé M., Mathieu R., Renard-Penna R. (2023). Micro-ultrasound-guided biopsies versus systematic biopsies in the detection of prostate cancer: A systematic review and meta-analysis. World J. Urol..

[32] Rojo E.G., Gómez B.G., Sutil R.S., Arzayus D.V., Quintas J.J., Barreras S.G., Menéndez R.B., Vallejo E.P., Montalvo C.C., Curtis D.L. (2024). Comparison in Detection Rate of Clinically Significant Prostate Cancer Between Microultrasound-guided Prostate Biopsy (ExactVu) and Multiparametric Resonance Imaging-guided Prostate Biopsy (Koelis System). Urology.

[33] Zhou S.R., Choi M.H., Vesal S., Kinnaird A., Brisbane W.G., Lughezzani G., Maffei D., Fasulo V., Albers P., Zhang L. (2024). Inter-reader Agreement for Prostate Cancer Detection Using Micro-ultrasound: A Multi-institutional Study. Eur. Urol. Open Sci..

[34] Grover V.P.B., Tognarelli J.M., Crossey M.M.E., Cox I.J., Taylor-Robinson S.D., McPhail M.J.W. (2015). Magnetic Resonance Imaging: Principles and Techniques: Lessons for Clinicians. J. Clin. Exp. Hepatol..

[35] Turkbey B., Brown A.M., Sankineni S., Wood B.J., Pinto P.A., Choyke P.L. (2016). Multiparametric prostate magnetic resonance imaging in the evaluation of prostate cancer. CA Cancer J. Clin..

[36] Barentsz J.O., Richenberg J., Clements R., Choyke P., Verma S., Villeirs G., Rouviere O., Logager V., Fütterer J.J. (2012). ESUR prostate MR guidelines 2012. Eur. Radiol..

[37] Weinreb J.C., Barentsz J.O., Choyke P.L., Cornud F., Haider M.A., Macura K.J., Margolis D., Schnall M.D., Shtern F., Tempany C.M. (2016). PI-RADS Prostate Imaging–Reporting and Data System: 2015, Version 2. Eur. Urol..

[38] Woo S., Suh C.H., Kim S.Y., Cho J.Y., Kim S.H. (2017). Diagnostic Performance of Prostate Imaging Reporting and Data System Version 2 for Detection of Prostate Cancer: A Systematic Review and Diagnostic Meta-analysis. Eur. Urol..

[39] Turkbey B., Rosenkrantz A.B., Haider M.A., Padhani A.R., Villeirs G., Macura K.J., Tempany C.M., Choyke P.L., Cornud F., Margolis D.J. (2019). Prostate Imaging Reporting and Data System Version 2.1: 2019 Update of Prostate Imaging Reporting and Data System Version 2. Eur. Urol..

[40] Park K.J., Choi S.H., Kim M.-H., Kim J.K., Jeong I.G. (2021). Performance of Prostate Imaging Reporting and Data System Version 2.1 for Diagnosis of Prostate Cancer: A Systematic Review and Meta-Analysis. J. Magn. Reason. Imaging.

[41] Kasivisvanathan V., Rannikko A.S., Borghi M., Panebianco V., Mynderse L.A., Vaarala M.H., Briganti A., Budäus L., Hellawell G., Hindley R.G. (2018). MRI-Targeted or Standard Biopsy for Prostate-Cancer Diagnosis. N. Engl. J. Med..

[42] Van Der Leest M., Cornel E., Israël B., Hendriks R., Padhani A.R., Hoogenboom M., Zamecnik P., Bakker D., Setiasti A.Y., Veltman J. (2019). Head-to-head Comparison of Transrectal Ultrasound-guided Prostate Biopsy Versus Multiparametric Prostate Resonance Imaging with Subsequent Magnetic Resonance-guided Biopsy in Biopsy-naïve Men with Elevated Prostate-specific Antigen: A Large Prospective Multicenter Clinical Study. Eur. Urol..

[43] Rouvière O., Puech P., Renard-Penna R., Claudon M., Roy C., Mège-Lechevallier F., Decaussin-Petrucci M., Dubreuil-Chambardel M., Magaud L., Remontet L. (2019). Use of prostate systematic and targeted biopsy on the basis of multiparametric MRI in biopsy-naive patients (MRI-FIRST): A prospective, multicentre, paired diagnostic study. Lancet Oncol..

[44] Prostate Cancer: Early Detection Guideline-American Urological Association. https://www.auanet.org/guidelines-and-quality/guidelines/prostate-cancer-early-detection-guideline?utm.

[45] Exact Imaging Announces Health Canada Approval and License for its ExactVu™ Micro-Ultrasound System for Prostate Imaging and Biopsy-Exact Imaging. https://www.exactimaging.com/press-releases/exact-imaging-announces-health-canada-approval-and-license.

[46] Urology of Virginia First US Practice to Acquire Novel ExactVu™ Micro-Ultrasound System for Prostate Imaging and Biopsy-Exact Imaging. https://www.exactimaging.com/press-releases/urology-of-virginia-first-us-practice-to-acquire-novel-exactvu-system.

[47] Ng A., Swanevelder J. (2011). Resolution in ultrasound imaging. Contin. Educ. Anaesth. Crit. Care Pain.

[48] Vassallo R., Mannas M.P., Salcudean S.E. (2025). Black PC. Developments in Ultrasound-Based Imaging for Prostate Cancer Detection. Prostate.

[49] PN 7095 PRI-MUS Anterior Quick Reference Poster EN Rev 1.2.pdf. https://www.exactimaging.com/images/manuals/English/PN%207095%20PRI-MUS%20Anterior%20Quick%20Reference%20Poster%20EN%20Rev%201.2.pdf.

[50] Schaer S., Rakauskas A., Dagher J., Rosa S.L., Pensa J., Brisbane W., Marks L., Kinnaird A., Abouassaly R., Klein E. (2023). Assessing cancer risk in the anterior part of the prostate using micro-ultrasound: Validation of a novel distinct protocol. World J. Urol..

[51] van Velthoven R., Diamand R., Mozer P., Barry de Longchamp N. (2024). Letter to the Editor on “Comparison in Detection Rate of Clinically Significant Prostate Cancer Between Microultrasound-guided Prostate Biopsy (ExactVu) and Multiparametric Resonance Imaging-guided Prostate Biopsy (Koelis System)”. Urology.

[52] Rosenkrantz A.B., Ayoola A., Hoffman D., Khasgiwala A., Prabhu V., Smereka P., Somberg M., Taneja S.S. (2017). The Learning Curve in Prostate MRI Interpretation: Self-Directed Learning Versus Continual Reader Feedback. Am. J. Roentgenol..

[53] Halstuch D., Baniel J., Lifshitz D., Sela S., Ber Y., Margel D. (2019). Characterizing the learning curve of MRI-US fusion prostate biopsies. Prostate Cancer Prostatic Dis..

[54] Kasabwala K., Patel N., Cricco-Lizza E., Shimpi A.A., Weng S., Buchmann R.M., Motanagh S., Wu Y., Banerjee S., Khani F. (2019). The Learning Curve for Magnetic Resonance Imaging/Ultrasound Fusion-guided Prostate Biopsy. Eur. Urol. Oncol..

[55] Cash H., Hofbauer S.L., Shore N., Pavlovich C.P., Bulang S., Schostak M., Planken E., Jaspars J.J., Luger F., Klotz L. (2022). Prostate Cancer Detection by Novice Micro-Ultrasound Users Enrolled in a Training Program. Société Int. D’Urologie J..

[56] University of Alberta (2025). A Phase 3, Multicenter, International, Non-Inferiority, Randomized Clinical Trial Comparing Screening for Prostate Cancer Using High Resolution Micro-Ultrasound Versus Multiparametric Magnetic Resonance Imaging (MUSIC-Screen).

[57] University of Alberta (2025). Micro-UltraSound in Cancer-Active Surveillance (MUSIC-AS).

[58] Regis F., Casale P., Persico F., Colombo P., Cieri M., Guazzoni G., Buffi N.M., Lughezzani G. (2020). Use of 29-MHz Micro-ultrasound for Local Staging of Prostate Cancer in Patients Scheduled for Radical Prostatectomy: A Feasibility Study. Eur. Urol. Open Sci..

[59] Fasulo V., Buffi N.M., Regis F., Paciotti M., Persico F., Maffei D., Uleri A., Saita A., Casale P., Hurle R. (2022). Use of high-resolution micro-ultrasound to predict extraprostatic extension of prostate cancer prior to surgery: A prospective single-institutional study. World J. Urol..

[60] Seaman E.K., Sawczuk I.S., Fatal M., Olsson C.A., Shabsigh R. (1996). Transperineal prostate needle biopsy guided by transurethral ultrasound in patients without a rectum. Urology.

[61] He Y., Liu X., Zhang J., Peng C. (2023). A Backing-Layer-Shared Miniature Dual-Frequency Ultrasound Probe for Intravascular Ultrasound Imaging: In Vitro and Ex Vivo Validations. Biosensors.

[62] Richemond A., Peters M., Schaer S., Dagher J., La Rosa S., Matthey J., Vietti-Violi N., Roth B., Lucca I., Valerio M. (2025). Predicting pathological tumor volume in prostate cancer lesions: A head-to-head comparison of micro-ultrasound vs. MRI. Urol. Oncol. Semin. Orig. Investig..

[63] Bhattacharya I., Khandwala Y.S., Vesal S., Shao W., Yang Q., Soerensen S.J.C., Fan R.E., Ghanouni P., Kunder C.A., Brooks J.D. (2022). A review of artificial intelligence in prostate cancer detection on imaging. Ther. Adv. Urol..

[64] Rohrbach D., Wodlinger B., Wen J., Mamou J., Feleppa E. (2018). High-Frequency Quantitative Ultrasound for Imaging Prostate Cancer Using a Novel Micro-Ultrasound Scanner. Ultrasound Med. Biol..

[65] Shao Y., Wang J., Wodlinger B., Salcudean S.E. (2020). Improving Prostate Cancer (PCa) Classification Performance by Using Three-Player Minimax Game to Reduce Data Source Heterogeneity. IEEE Trans. Med. Imaging.

[66] Jiang H., Imran M., Muralidharan P., Patel A., Pensa J., Liang M., Benidir T., Grajo J.R., Joseph J.P., Terry R. (2024). MicroSegNet: A deep learning approach for prostate segmentation on micro-ultrasound images. Comput. Med. Imaging Graph..

